# Histone and non-histone lactylation: molecular mechanisms, biological functions, diseases, and therapeutic targets

**DOI:** 10.1186/s43556-025-00275-6

**Published:** 2025-06-09

**Authors:** Xia Peng, Juan Du

**Affiliations:** 1 Department of Geriatric Dentistry, Capital Medical University School of Stomatology, Fanjiacun Road No.9, Beijing, 100070 China; 2 Laboratory of Orofacial Development, Laboratory of Molecular Signaling and Stem Cells Therapy, Molecular Laboratory for Gene Therapy and Tooth Regeneration, Beijing Key Laboratory of Tooth Regeneration and Function Reconstruction, Capital Medical University School of Stomatology, Beijing, Fanjiacun Road No.9, 100070 China

**Keywords:** Lactylation (Kla), Histone, Non-Histone, Post-Translational Modification

## Abstract

Lysine lactylation (Kla) is a recently discovered post‑translational modification in which a lactyl moiety is transferred onto the ε‑amino group of lysine residues, linking cellular metabolism to epigenetic and signaling pathways. This process is regulated by a range of enzymes and metabolites, including lactate, “lactyltransferases (writers)”, “Delactylases (erasers)”, and “readers” involved in the modification. Histone lactylation has been observed in H2A, H2B, H3, and H4, with H3K18la and H4K12la being the most extensively studied sites, linked to numerous biological functions. Beyond chromatin, Kla has also been identified in a growing number of non-histone proteins, further expanding its functional significance. For instance, non-histone proteins such as AARS1-K120la, ACSS2-Kla, MRE11-K673la, NBS1-K388la and GNAT13-Kla has illuminated novel regulatory mechanisms and reinforced the potential of non-histone Kla as a promising avenue for research. Importantly, aberrant Kla patterns have been linked to various disease states, including cancer, inflammation, and metabolic disorders, highlighting its emerging potential as a biomarker and therapeutic target. In this review, we systematically summarize the molecular mechanisms, biological functions, disease associations, and therapeutic implications of both histone and non-histone Kla. By integrating current findings and discussing existing challenges, we aim to provide a comprehensive overview that will deepen understanding of Kla biology and inspire future research into its diagnostic and therapeutic potential.

## Introduction

Post-translational modifications (PTMs), serving as pivotal regulatory mechanisms governing protein functional diversity, manifest through enzymatic catalysis at distinct amino acid side chains [1]. Given the pronounced chemical reactivity of lysine ε-amino groups, lysine acylation emerges as the most ubiquitous form of PTM [2]. Canonical lysine acylation encompasses acetylation, phosphorylation, methylation, glycosylation, and ubiquitination. Contemporary investigations have further elucidated that diverse metabolic intermediate, including lactate, succinate, butyryl-CoA, and crotonyl-CoA, can function as acylation substrates, which are referred to as novel lysine acylation modifications [3–5].

Lysine lactylation (Kla), the seminal breakthrough in novel lysine acylation modifications, was first characterized through pioneering work by Zhang et al. in 2019 [6]. Their groundbreaking revelation established lactate’s capacity to serve as a novel substrate for lysine acylation modifications, exerting direct regulatory influence on downstream gene transcription. This landmark discovery further delineated distinct temporal dynamics differentiating Kla from lysine acetylation (Kac), providing crucial mechanistic insights for elucidating lactate’s multifaceted roles across diverse pathological contexts.

Kla occurs in both histone and non-histone proteins, with histone Kla constituting the most extensively characterized subtype. This modification participates in fundamental physiological processes, including pre-implantation embryo development, oocyte meiosis, neurodevelopment and so on [7–9]. Pathologically, Kla has been implicated in spanning nitrosamine-induced pulmonary fibrosis (As-IPF), colorectal cancer, lung adenocarcinoma, prostate cancer (PC), Alzheimer's disease, mechanical pain, osteoporosis, sepsis, hypoxic pulmonary hypertension, septic shock and so on [10–15]. Notably, Kla demonstrated paradoxical regulatory duality in oncogenesis, mechanistically influencing angiogenesis through VEGF/FGF signaling, modulating tumor immunity *via* CD8^+^ T cell dynamics, and driving proliferation through β-catenin/KCNK1 pathways. Therapeutic exploration revealed Kla’s clinical potential, exemplified by synergistic tumor suppression combined with anti-PD-1 immunotherapy. However, there are still lot of unknown knowledge about lysine Kla: undefined L-lactyl-CoA biosynthesis mechanisms, unresolved enzymatic versus non-enzymatic lactylation catalysis, ambiguous substrate specificity for lactate stereoisomers (L-lactate/D-lactate), and uncharacterized crosstalk between specific histone Kla sites (e.g., H3 K18 la) and other PTMs. These unresolved questions underscore the imperative for systematically investigating Kla’s molecular mechanism.

Emerging proteomic advancements have propelled extensive investigation into non-histone Kla [16]. The Kla profiling study in hepatocellular carcinoma identified 9,275 Kla sites, with 9,256 non-histone sites detected [17]. Despite its recent discovery, non-histone Kla has demonstrated pathophysiological relevance in atherosclerosis, inflammatory responses, lung cancer, pulmonary fibrosis, liver injury, and retinopathy [18–24]. Moreover, its therapeutic potential has been validated. Pharmacological targeting of the RIG-I-Kla site suppressed colorectal cancer proliferation, while ACSS2-KAT2A interaction enhanced anti-PD-1 immunotherapy efficacy. Notably, non-histone AARS1 mediated cGAS-Kla to modulate innate immunity through lactate sensing. Intriguingly, the non-enzymatic S-D-lactoylglutathione (SLG)-dependent Kla reaction identified *via* D-lactate and the critical role of acetyl-CoA synthetase 2 (ACSS2) in L-lactyl-CoA biosynthesis have been recently uncovered in studies exploring non-histone Kla [25, 26]. These findings represented initial insights into non-histone Kla’s functional diversity, suggesting broader regulatory roles awaiting discovery.

Based on this, this review summarized the origin and biological process of Kla and the latest Kla detection techniques. We provided a comparative overview of Kla sites, molecular mechanisms, disease models, and cellular functions between histone and non-histone proteins. In addition, we highlighted PTMs that crosstalk with Kla, described in detail the physiological and pathological processes regulated by Kla, and summarized current therapeutic targets. We hope this review offers researchers a comprehensive understanding of Kla and serves as a valuable resource to support their future studies.

## Molecular mechanisms of lysine lactylation

**Fig. 1:**
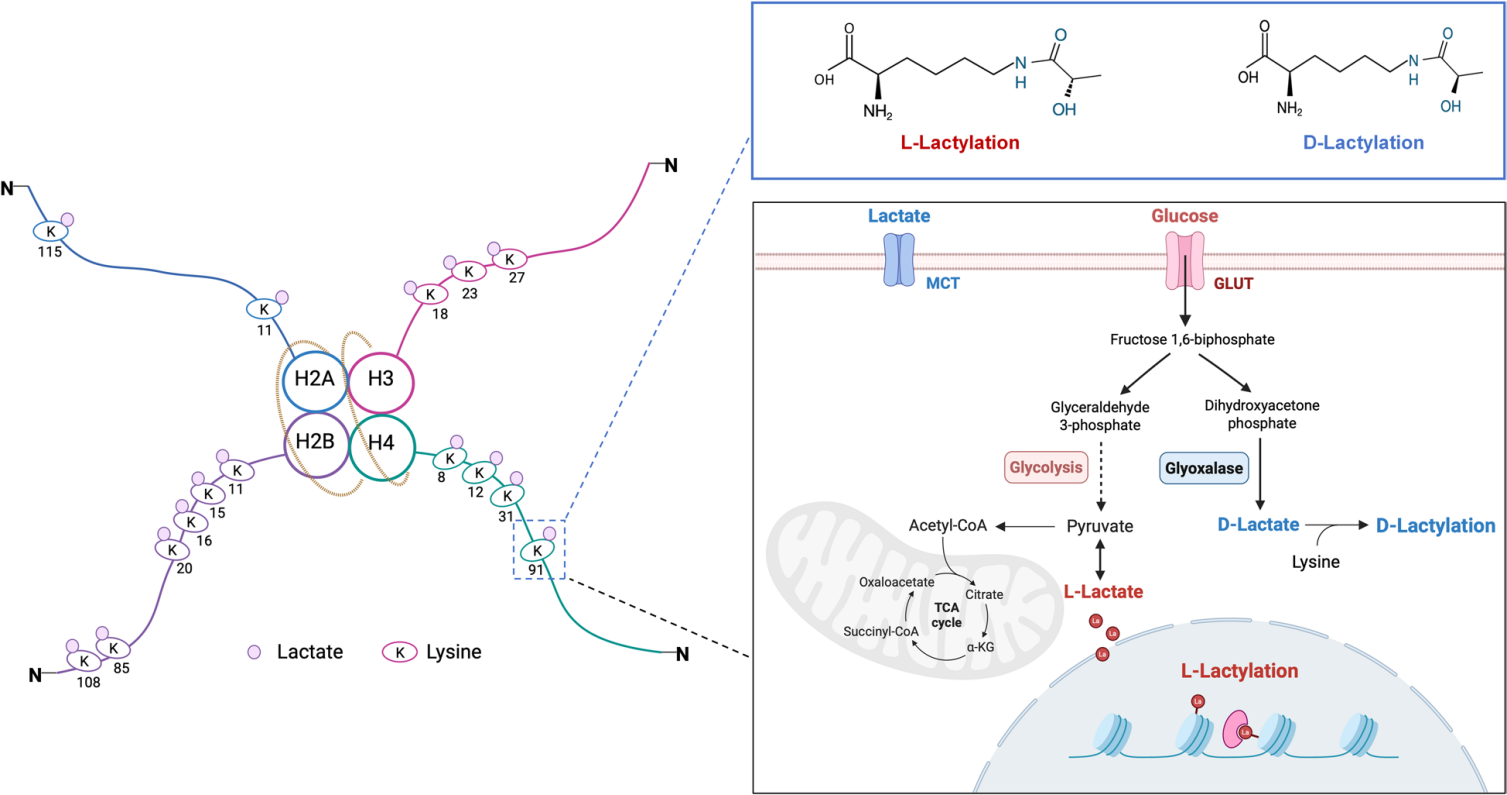
Sites of histone lysine lactylation. This figure illustrates the major lysine lactylation sites and processes identified to date. H2 A, H2B, H3, and H4 represent the four simplified histone types, and K denotes lysine residues. The left panel shows the specific lactylation sites on H2A, H2B, H3, and H4. The upper right section shows the two recently identified forms of lactylation, with K_L-la_ on the left and K_D-la_ on the right. The lower right section depicts how L-lactate derived from glycolysis and D-lactate produced *via* the glyoxalase pathway respectively activate K_L-la_ and K_D-la_

**Fig. 2:**
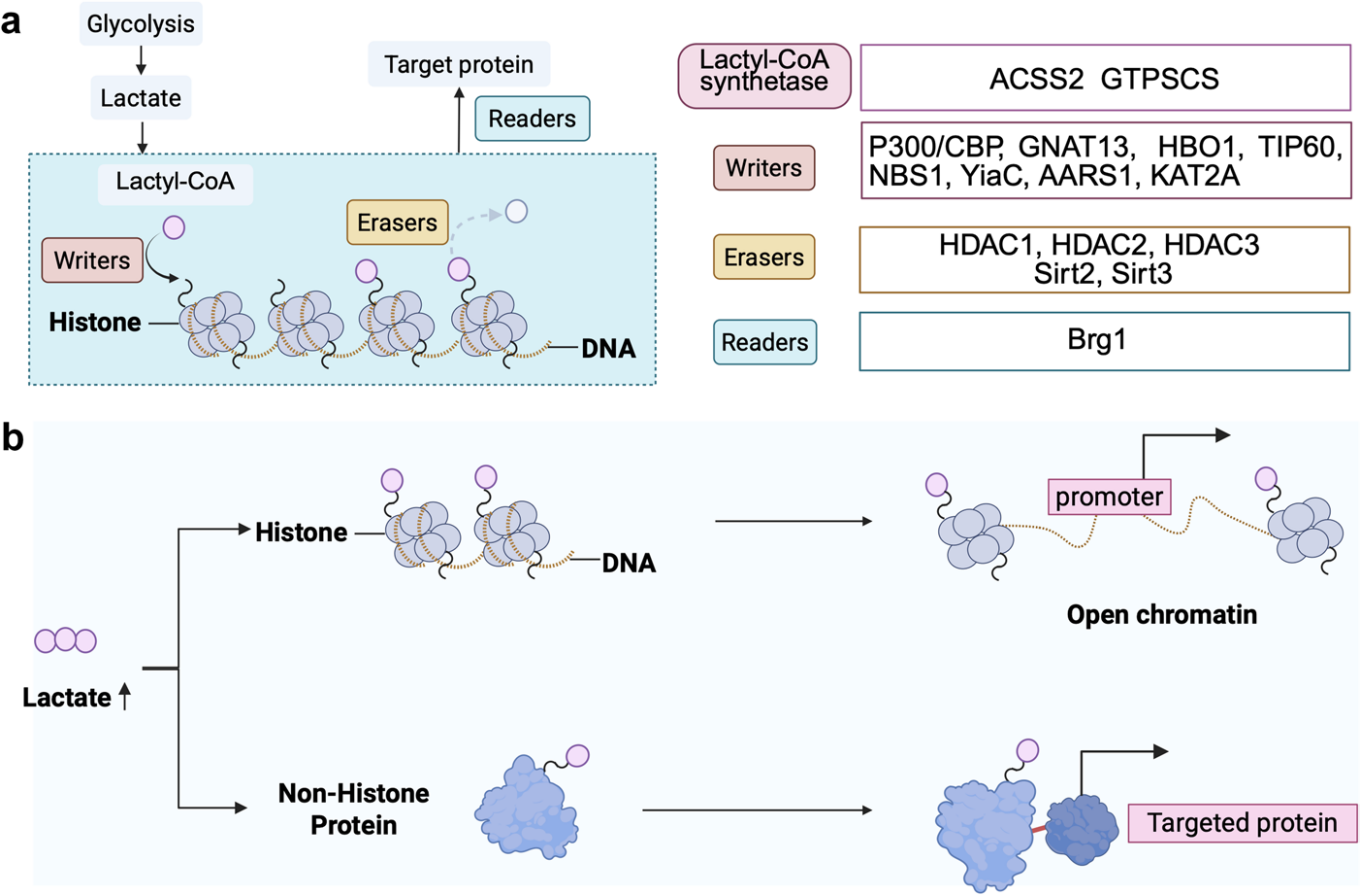
The process of histone lactylation and non-histone lactylation.** a** Regulation of lysine lactylation. Cells produce lactate upon various stimulations. The lactate then acts as a substrate for lactylation. The addition of lactate to histone lysine residues is mediated by enzymes known as “writers”, “erasers” and “readers”. **b** The difference of histone and non-histone lysine lactylation. Increased lactate levels lead to the lactylation of histone proteins, which subsequently affects chromatin structure and gene expression. Non-histone lactylation alters protein function by changing the spatial conformation of target proteins

The establishment of Kla followed these essential steps: the accumulation of lactate is a crucial prerequisite for initiating Kla, serving as a key trigger for this modification, whether it occurs on histones or non-histone proteins [27]. The extent of Kla is positively correlated with intracellular lactate levels [28]. However, lactate cannot directly serve as the donor for Kla, and it must first be converted into L-lactyl-CoA through an unknown pathway (Fig. 1) [29]. Then, Kla represents a dynamic reversible modification, where acyltransferases function as “writers” and deacetylases serve as “erasers”. Under the action of “writers”, L-lactyl-CoA is transferred to histones or non-histone proteins in the nucleus, initiating Kla. Conversely, histone deacetylases can remove L-lactyl-CoA from histones or non-histone proteins, interrupting the Kla process (Fig. 2a) [30]. Finally, readers are responsible for recognizing and interpreting Kla, transmitting signals to downstream targets. As demonstrated by the preceding evidence, Kla is regulated by multiple enzymes and metabolites, while each regulatory step involves more intricate mechanisms.

### Role of lactate metabolism and substrate supply

Lactate exists in two isomeric forms: L-lactate and D-lactate [31–34]. L-lactate is produced via pyruvate, whereas D-lactate arises as a byproduct of methylglyoxal (MGO), which is formed through the aldehyde reductase pathway during glycolysis [35]. The conjugation of lactate with lysine generates three structural isomers: K_L-la_, K_D-la_, and K_ce_. Conventional high-performance liquid chromatography (HPLC) fails to differentiate these isomers, leading to persistent ambiguity regarding Kla’s biochemical origins [36]. Zhao et al. resolved this challenge by employing multiple orthogonal techniques (integrating analytical, chemical biological, and immunological approaches) to distinguish these structural variants. Their work definitively demonstrated that K_L-la_ formation occurs exclusively through glycolytically derived L-lactate, thereby ushering in a new era of precision for K_L-la_ research. Recent evidence demonstrated that D-lactate can drive Kla through an SLG-dependent non-enzymatic mechanism in inflammatory disorders [25]. Elucidating the mechanistic basis of K_D-la_ and its pathophysiological roles may enable precise modulation of inflammatory responses, offering novel therapeutic strategies for inflammation-associated diseases. Moreover, this established that K_L-la_ is a metabolism-related enzymatic reaction occurring in the nucleus with L-lactate as the substrate, whereas K_D-la_ is an immune-related non-enzymatic reaction occurring in the cytoplasm with D-lactate as the substrate.

### L-lactyl-CoA synthetases

Following lactate generation in the cytoplasm, its conjugation with lysine requires prior conversion to L-lactyl-CoA. The enzyme responsible for this critical step remained unidentified until recent investigations in EGFR-activated cancer cells [37]. Zhu et al. demonstrated that ACSS2 functions as an L-lactyl-CoA synthetase [26]. They showed that blocking the ACSS2-KAT2 A interaction with a peptide, combined with anti-PD-1 antibodies, significantly enhanced tumor suppression. However, the authors noted that this ACSS2-KAT2 A interaction primarily occurs at H3 K14 la and H3 K18 la. Meanwhile, Zhang et al. also identified GTP-specific SCS (GTPSCS) as a key L-lactyl-CoA synthetase in the nucleus. They demonstrated that when both L-lactyl-CoA and succinyl-CoA were added, GTPSCS predominantly enhanced histone Kla rather than Lysine Succinylation (Ksucc). The interaction between GTPSCS and p300 regulated H3 K18 la and Growth differentiation factor 15 (GDF15) expression, promoting glioma proliferation and radio resistance.

### Enzymatic machinery

#### Lactyltransferases (Writers)

The currently known “writers” of Kla are E1A-associated protein p300/CREB-binding protein (p300/CBP), Gcn5-related N-acetyltransferase 13 (GNAT13), Histone acetyltransferase binding to ORC1 (HBO1), YiaC, TIP60, NBS1, Alanyl-tRNA synthetase 1 (AARS1), and Lysine acetyltransferase 2 A (KAT2A). Among them, p300/CBP was the first to be identified. In 2019, Zhang et al. confirmed the existence of histone Kla and identified p300 as potential “writer”. Using L-lactyl-CoA instead of acetyl-CoA, they showed a high dependence of H3Kla and H4Kla on P53 and p300, like acetylation. Knockdown of p300 in HCT116 and HEK293T cells resulted in decreased Kla levels, while overexpression of p300 in HEK293T cells increased Kla levels, confirming p300 as a potential “writer”. However, the authors also noted that the indirect roles of p300 could not be ruled out, necessitating further research. Niu et al. also discovered that HBO1, a member of the KAT family, functions as a “writer” for Kla, with a particular preference for catalyzing histone H3K9la [38, 39]. This finding fills an important gap, shedding light on the role of MYST enzymes within the KAT family in Kla regulation. Additionally, Dong et al. identified YiaC from the GNAT family of N-acetyltransferases in *Escherichia coli* as a “writer”, while CobB was characterized as an eraser primarily associated with metabolic processes [40]. Similarly, in the cariogenic bacterium *Streptococcus mutans*, Li et al. discovered that GNAT13, an enzyme from the GNAT superfamily, acts as the “writer” for Kla at K173 of RNA polymerase subunit α (RpoA). Overexpression of GNAT13 was found to inhibit biofilm formation, hinting at its potential therapeutic implications [41].

Moreover, Chen et al. linked NBS1 to chemotherapy responses and DNA repair in cancer. Their research demonstrated that TIP60 functions as the “writer” for NBS1-K388la, while HDAC3 serves as its “eraser”. Inhibiting NBS1-K388la decreased DNA repair efficiency and overcame chemotherapy resistance, providing a crucial new perspective for cancer treatment strategies [42]. Importantly, Xie et al. revealed that targeting “writers” could represent a novel strategy for cancer therapy. They focused on eEF1A2, a protein that regulates tumor progression *via* PTM, which led to identifying KAT8 as the “writer” responsible for eEF1A2-Kla. In a high-lactate-induced Colorectal Cancer (CRC) mouse model, KAT8’s tumor-suppressive role was validated, offering new insights into potential therapeutic targets [43].

However, “writers” identified in these studies are based on L-lactyl-CoA as the substrate for the modification. Varner’s study in 2020 using liquid chromatography-mass spectrometry to analyze L-lactyl-CoA concentrations in cells and tissue found that the L-lactyl-CoA levels are 20 to 350 times lower than those of other major acyl-CoA molecules like acetyl-CoA, propionyl-CoA, and succinyl-CoA [44]. The enzyme responsible for producing L-lactyl-CoA remains unidentified. Additionally, Ju et al. proposed that the Kla process may differ from acetylation and might not necessarily rely on the transport of L-lactyl-CoA. They suggested that metabolic changes, including shifts in lactate and ATP levels, might enable enzymes such as AARS1 to directly transport lactate into the nucleus, where it could interact with downstream target genes like YAP-TEAD, driving the modification [45]. However, these hypotheses remain unresolved and represent key areas for further investigation in future research.

#### Delactylases (Erasers)

The erasers for Kla are currently thought to be HDAC1, HDAC2, HDAC3, SIRT2 and SIRT3 [46, 47]. Moreno-Yruela found that Kla is enzymatically driven rather than a spontaneous chemical reaction. Like other short-chain acylation modifications, Kla was dynamically regulated by HDACs, with nuclear HDAC1-3 being responsible for the reversible modulation of Kla. Meanwhile, they found that HDACs preferentially target the H4K5la, though the reasons for this specificity remain unknown [47]. Fan et al. observed that SIRT3 exhibits markedly enhanced erasure activity at the H4K16la site compared to other human sirtuins [46].

Regarding the therapeutic potential of erasers, Sun et al. discovered that combining Sirt2 inhibitors with copper ion carriers, such as Elesclomol, effectively address the challenges posed by elevated copper and acidity levels in gastric cancer (GC) [48].

#### Readers

Limited research has been done on readers involved in Kla. However, Hu et al. revealed that Dux, a key factor regulating the pluripotency of induced pluripotent stem cells (iPSCs), regulated iPSC metabolic reprogramming through H3K18la. Further experiments demonstrated that both H3K18la and Brg1 are enriched at the promoters of genes associated with pluripotency and epithelial adhesion, indicating that Brg1 is the reader [49].

## Detection techniques of lysine lactylation

The discovery of Kla is closely tied to advancements in technology. In particular, the identification of Kla has been made possible by the development of mass spectrometry (MS) techniques. In 2019, Zhang et al. employed isotope tracing combined with tandem MS (MS/MS) to identify Kla sites. Since then, numerous studies used similar mass spectrometry approaches for detection. However, Wan et al. pointed out that traditional PTM identification relies on the fixed mass shifts of peptide precursors and MS/MS fragment ions, which can lead to false positives. They emphasized the necessity of identifying specific MS/MS ions for Kla. Their research discovered that during tandem MS analysis, the cyclic ammonium ions formed by lactylated lysine could reliably facilitate protein Kla mapping. The Cyclm ion in Kla exhibited a strong affinity for Kla spectra, suggesting that the inclusion of this ion in Kla identification standards should be considered [50].

Moreover, Kla research greatly benefits from the development of high-throughput technologies. CUT&Tag (Cleavage Under Targets and Tagmentation), a novel technique for studying DNA-protein interactions, represents an upgraded version of ChIP-seq, offering advantages such as high resolution, low cell input, and an efficient, straightforward protocol [51, 52]. CUT&Tag has played a key role in exploring Kla sites and is often combined with RNA-seq analysis to identify downstream targets. This combination has been successfully validated in a range of conditions, including liver fibrosis, myocardial infarction, bladder cancer, osteogenesis, viral replication, autoimmune uveitis, breast cancer, and ectodermal differentiation [22, 53–57]. However, CUT&Tag still has some limitations, primarily relying on the precision of antibodies, which may affect result accuracy. Interestingly, Bárcenas-Walls et al. from Sweden discovered a new approach: a single-cell CUT&Tag (Nano-CT) based on nanobodies. This approach facilitated the concurrent analysis of two histone modifications and chromatin accessibility within the same single cell [58]. It’s anticipated that more innovative techniques will emerge in the future to assist in Kla detection.

## Histone and non-Histone Lactylation Dynamics

The advancement of high-throughput proteomics has facilitated the systematic identification and investigation of non-histone Kla sites. Although non-histone Kla shares activation mechanisms with histone Kla, significant distinctions exist between these two modification types (Fig. 2b) (Table 1) [59, 60]. Firstly, histone Kla predominantly occurs on core histones (H2 A, H2B, H3, and H4), whereas non-histone Kla primarily targets enzymes, transcription factors, and DNA damage repair proteins. Additionally, the number of modification sites differs substantially. Current literature reported over 9,000 non-histone Kla targets, compared to merely 28 sites in histones due to spatial constraints of nuclear architecture. Furthermore, the mechanistic divergence is reflected in reaction specificity. Histone Kla relies solely on L-lactate-driven enzymatic activity, while non-histone modifications can be catalyzed by both L-lactate and D-lactate through enzymatic or non-enzymatic routes. In terms of functional mechanisms, histone Kla regulates chromatin topology to modulate transcriptional accessibility, in contrast to non-histone Kla, which predominantly alters enzymatic activity, protein stability, and interactome remodeling [61]. Regarding biological processes, histone modifications are closely associated with DNA compaction and transcriptional regulation, whereas non-histone Kla participates in dynamic cellular processes including signal transduction, metabolic reprogramming, and cell cycle control. This comprehensive analysis of Kla dynamics not only deepens our understanding of fundamental biological regulation but also highlights potential therapeutic targets for diseases linked to aberrant post-translational modifications.

**Table 1:** Comparison of Histone Lactylation and non-Histone Lactylation

**Aspect**	**Histone lactylation**	**non-histone lactylation**
Target Proteins	Primarily occurs on histones H2A, H2B, H3, and H4	Found on various enzymes, transcription factors, and DNA damage repair proteins
Number of Modification Sites	Limited (28 sites) due to structural constraints	Exceeds 9,000 lactylation sites according to current literature
Type of Reaction	Enzymatic reaction induced by L-lactate	Enzymatic or non-enzymatic reactions induced by L-lactate or D-lactate
Functional Mechanism	Alters chromatin conformation to regulate gene transcription	Modulates enzymatic activity, affects protein function, and reshapes protein interaction networks
Biological Significance	Primarily involved in epigenetic regulation	Implicated in diverse cellular processes such as metabolism, DNA repair, and signal transduction

### Histone lysine lactylation dynamics

Histone Kla is the Kla modification occurring on the lysine residues of histones, and it is a novel type of post-translational modification. Its functions vary depending on the location of the lysine residues within different histones, classified into H2A, H2B, H3, and H4. In 2019, Zhang et al. identified 28 common Kla sites in human HeLa cells and mouse BMDM cells, including common H2AK11la, H2AK115la, H2BK11la, H2BK15la, H2BK16la, H2BK20la, H2BK85la, H2BK108la, H3K18la, H3K23la, H3K27la, H4K8la, H4K12la, H4K31la, and H4K91la (Fig. 1). These modifications have been validated to exert functional roles in more than 20 different cell types. For example, Galle et al. compared the genome-wide distribution of H3K18la in various samples (mouse embryonic stem cells, macrophages, adipocytes, and mouse and human skeletal muscles) [62]. In addition, the range of target genes is remarkably diverse, involving Ythdf1, SLC25A29, ALKBH3, ARG1, TLR4, RUBCNL, and CCNB1 [63–69]. Among the identified Kla sites, H3K18la, H3K23la, and H4K12la have been studied most extensively. A summary of associated cell types, functions, disease contexts, and target genes is presented in Table 2.

**Table 2:** Histone lactylation in disease models and cellular functions

**Sites**	**Cell Type**	**Species**	**Disease Model**	**Target Gene**	**Reference**
H3K18la	Oocytes and pre-implantation embryos at different stages	Mouse	Pre-implantation embryo development	NA	[8]
	hypoxic pulmonary artery smooth muscle cell (PASMC)	Rat	ROS-mediated hypoxic pulmonary hypertension	HIF-1α targets (Bmp5, Trpc5, and Kit)	[10]
	Bone marrow mesenchymal stem cells	Mouse	Osteoporosis / Differentiation of bone mesenchymal stem cells (positively correlated)	Sequencing analysis of osteogenic genes COL1A2, COMP, ENPP1, and TCF7L2	[14]
	In vivo and in vitro multi tissue	Mouse	Development and mitosis related	Positively correlated with H3K27ac and H3K4me3	[62]
	MLE12 cells	Mouse	Arsenite-related idiopathic pulmonary fibrosis	Promotes Ythdf1 transcription, causing increased NREP expression	[63]
	Primary lung adenocarcinoma and adjacent normal samples	Human	Proliferation and migration of lung adenocarcinoma endothelial cells	Regulation of solute carrier (SLC25A29) promoter region	[64]
	human UM cell lines	Human	human ocular melanoma tissues and human normal nevus tissues	Promotes ALKBH3 gene transcription	[65]
	Peritonitis animal model	Mouse	Sepsis caused by drug-resistant infection(anti-MRSA)	Promotes Arg1 expression	[66]
	HCT116 cell lines	Mouse	Hypoxic metastatic colorectal cancer	Promotes RUBCNL/Pacer transcription	[67]
	Critically ill patients / healthy subjects	Human	Critically ill patients (septic shock / infection)	Positively correlated with Arg1 mRNA levels	[68]
	PC9-BrM3 cells	Mouse	non-small cell lung cancer (NSCLC)	Promotes CCNB1 transcription	[69]
	Colorectal cancer cells	Mouse	Liver metastasis of colorectal cancer	Promotes expression of chemokines CXCL1, CXCL5	[70]
	E13.5 telencephalon tissue	Mouse	Neurodevelopment	Promotes multiple genes transcription	[109]
	senescent BV2 cells (DoxBV2) and young BV2 cells (NC-BV2)	Mouse	Alzheimer's disease	Binds to Rela (p65) and NFkB1 (p50) promoter	[147]
	LLC, B16F10, and MC38 tumor cells	Human/Mouse	AOM/DSS colon cancer model	Promotes Mettl3 transcription	[170]
	Prostate cancer cells	Human	Angiogenesis in prostate cancer	Potential regulation of Sema3A transcription	[212]
H3K23la	oocytes	Mouse	mouse GV-stage oocytes	NA	[7]
	5XFAD cells / Microglia	Human	5XFAD Mouse	Promotes HIF1-α, PKM2 and LDHA transcription	[11]
H4K12la	8503c cell line	Human	Undifferentiated thyroid carcinoma (ATC)	Promotes CDK1, CCNE1 and AURKB transcription	[183]
	microglia	mouse	Spinal cord injury	Promotes PD-1 transcription	[195]

**Fig. 3:**
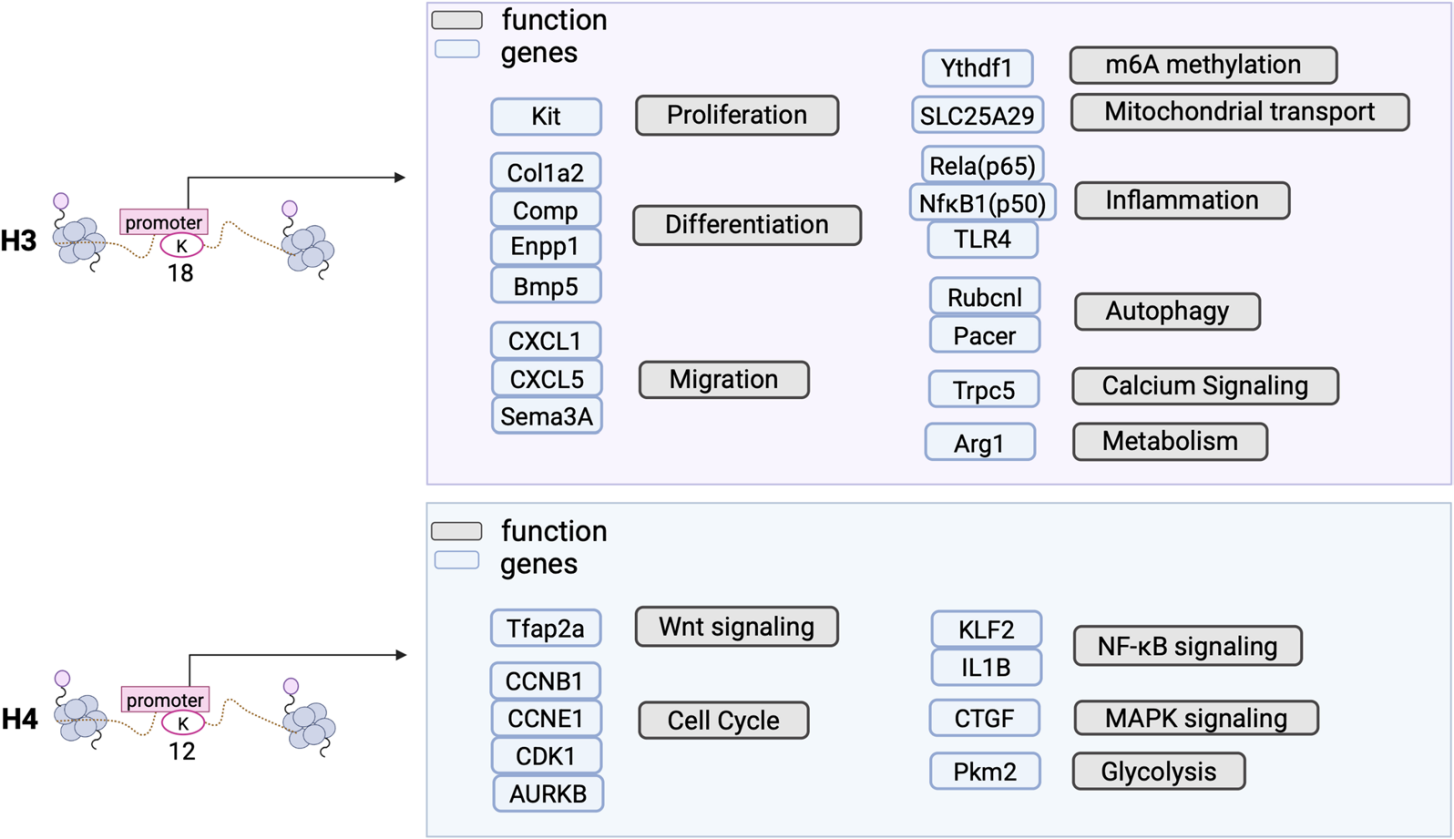
Biological function of histone H3K18la and Histone H4K12la. H3K18la and H4K12la are the two most important sites in histone lactylation. The target genes they regulate are involved in various essential aspects of cellular life. This figure summarizes the target genes associated with these two sites and their key functions

Histone Kla primarily triggers downstream chromatin remodeling, which directly leads to the activation or repression of target gene transcription. Current research primarily focused on H3K18la, H3K23la, and H4K12la (Fig. 3). H3K18la, a classic Kla site, has been identified as a key player in the transcriptional activation of numerous genes. For example, H3K18la influenced IPF by mediating gene expression, altered histone Kla dynamics in cancer progression, and drove macrophage polarization in methicillin-resistant Staphylococcus aureus (MRSA) infections [14, 68, 70]. Currently, H3K23la is primarily believed to be associated with mouse zygote development and ulcerative colitis (UC)[71]. Moreover, emerging evidence points to H4K12la-regulated genes involving cancer progression, chemoresistance, microglial repair, decidualization, microglia-mediated tissue repair processes, and mouse oocyte development [71, 72].

### Non-histone lysine lactylation dynamics

Compared to histones, Kla in non-histone proteins refers to the Kla modification occurring on the lysine residues of non-histone proteins. As mentioned above, sequencing analyses in HCC and KC have identified a substantial number of non-histone Kla sites. Due to the large number of non-histone proteins involved, non-histone Kla appears to affect a broader range of proteins and regulatory pathways. Moreover, unlike the direct Kla of lysine residues on histones, which primarily induces conformational changes, Kla on non-histone proteins mainly functions by enhancing their interactions with specific target proteins.

**Fig. 4:**
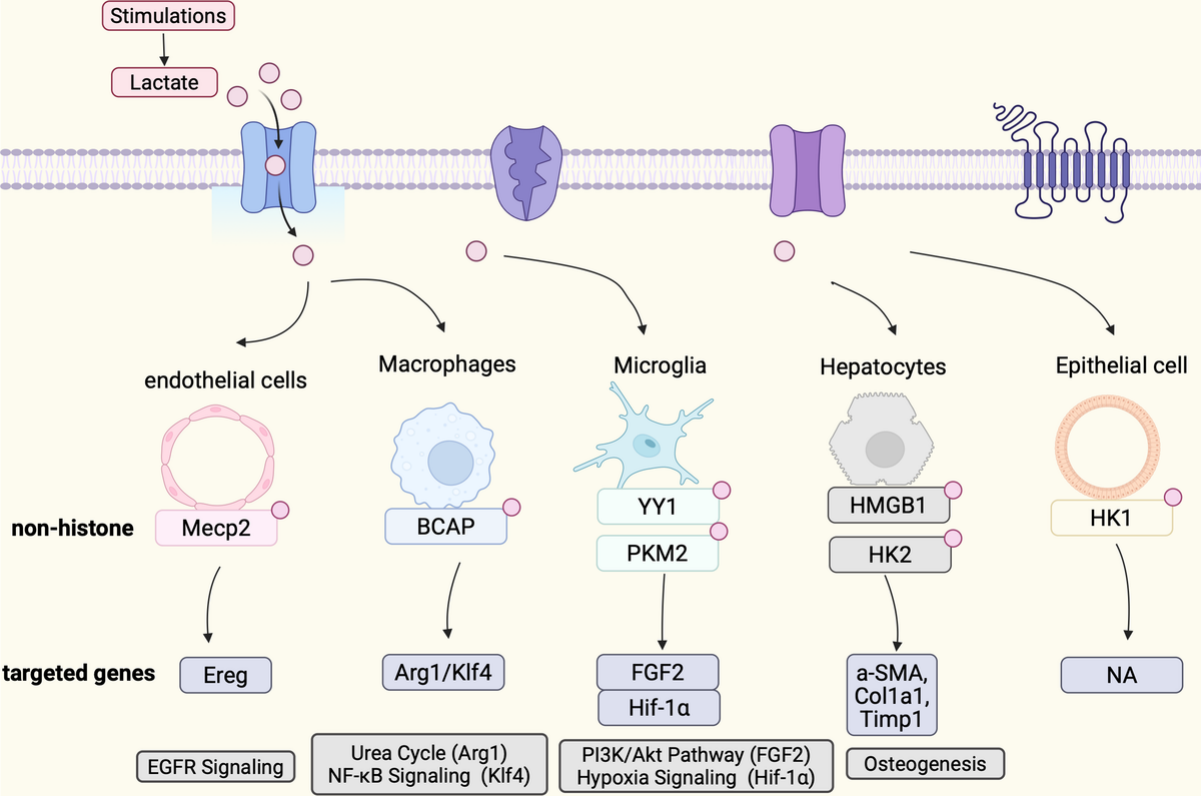
Biological function of non-histone lactylation. Non-histone proteins are more diverse than histones, and the cell types and biological functions they regulate are broader. We categorized by cell type and annotated the functional changes induced by non-histone lactylation in different cell types

Non-histone protein Kla is emerging as a critical regulator in various biological processes and diseases, paralleling the significance of histone Kla. Recent studies have uncovered its significant impact on various cell types (Fig. 4)(Table 3). To be specific, the Kla of proteins such as Vps34, Transcription Factor EB (TFEB), Discoidin, CUB, and LCCL domain-containing 1 (DCBLD1), Tau, NEDD4, Caspase-11, Gasdermin D (GSDMD), and NLR Family Pyrin Domain Containing 3 (Nlrp3) is associated with processes like autophagy, ferroptosis, and pyroptosis [73, 74]. Pyruvate Kinase M2 (PKM2) and Hexokinase (HK) are influenced by Kla, affecting cellular metabolism and disease states. Kla in proteins like YY1 and BCAP is linked to diseases such as cancer.

**Table 3:** Non-histone lactylation in disease models and cellular functions

**Non-Histone**	**Lactylation sites**	**Target Genes**	**Disease**	**Cell Type**	**Species**	**Reference**
PKM2		COL1A2, COMP, ENPP1, and TCF7L2	Pkm2⁻/⁻ model	Bone marrow endothelial cells	Mouse	[14]
Mecp2	K271	Ereg	Atherosclerosis	Mouse aortic endothelial cells (MAEC)	Mouse	[19]
Vps34	K356/K781	Beclin1, Atg14, and Uvarg	Lung cancer	H1299 cells, HEK293T cells, HeLa cells, and U2OS cells	Human	[21]
HK2	-	a-Sma, Col1a1, and Timp1	Liver fibrosis model	Hepatic stellate cells	Chinese hamster	[22]
HMGB1	-	NA	Liver hypoxia/reperfusion injury	Hepatocytes	Mouse	[23]
YY1	K183	FGF2	Retinal neovascular disease	Microglia	Mouse	[24]
DCBLD1	K172	G6PD	Cervical cancer	cervical cancer cells	Human	[73]
NEDD4	K33	Caspase-11	Acute liver injury	L929, HEK293, HEK293T cell lines	Mouse	[74]
Mettl3	-	TFRC	Intracerebral hemorrhage(ICH)	PC12 cell line	Rat	[106]
TFEB	K91	WWP2	Pancreatic cancer	PANC cellHela cell	Human	[110]
Tau	K677	NCOA4, FTH1	Alzheimer's disease	BV-2 cell	Mouse	[114]
HK1	H4K8la	NA	Non-small cell lung cancer model	BEAS-2B bronchial epithelial cell line and NSCLC cell lines A549 and H1299	Human	[130]
PKM2	K62	ARG1	LPS-induced inflammation model	BMDM	Mouse	[137]
BCAP	-	Arg1, Klf4	Reparative macrophages	Macrophages	Mouse	[152]
RIG-I	-	Nlrp3	Liver metastasis of colorectal cancer	MC38 cell	Mouse	[171]
ALKBH5	K284	ESCO2	Innate immunity	-	-	[172]
NLRP3	K245	LDHA	Ischemia/reperfusion(I/R)	the lung bronchial epithelial and macrophages	Human	[223]

Non-histone Kla extensively participates in various physiological and pathological processes by altering the activity, stability, subcellular localization, and protein interaction capacity of non-histone proteins. The lactate-driven ABCF1-K430la modification promoted its nuclear translocation, thereby activating the KDM3A-H3K9me2-HIF1α signaling axis, which serves as a representative example of histone Kla-mediated regulation of subcellular localization [75]. The activity of lactylated pyruvate dehydrogenase (PDHA1) is suppressed, leading to reduced acetyl-CoA production and indirectly regulating the interaction network of mitochondrial-related proteins, which demonstrates non-histone Kla on the protein network [76]. Moreover, non-histone Kla can also influence DNA repair and contribute to chemotherapy resistance. For instance, MRE11-K673la promoted DNA binding and enhanced homologous recombination repair, leading to tumor cell resistance to cisplatin and PARP inhibitors [77]. In addition, NBS1-K388la maintained the stability of the MRN complex, dynamically regulating repair efficiency [42].

## The crosstalk between lysine lactylation and other PTMs

The functional diversity of proteins critically depends on PTMs, where chemical groups are dynamically added to or removed from amino acid side chains or backbones through enzymatically regulated reversible processes [78]. A canonical example of PTM crosstalk occurs in the MAPK signaling pathway, where the phosphokinase cascade (*Ras*-*Raf*-*Erk* axis) enables rapid signal propagation through coordinated modification events [79]. Notably, during the initial characterization of Kla, Zhang et al. observed divergent modification patterns between Kla and Kac at identical residues following inflammatory stimulation, suggesting competitive occupancy of shared lysine residues by distinct PTM types. Therefore, exploring the relationship between Kla and other PTMs is essential for identifying Kla’s unique characteristics. Current literature indicated associations between Kla and m^6^A methylation, acetylation, phosphorylation, and crotonylation, suggesting that multiple modifications can synergize to help cells adapt to changing environments [80–82]. Here, we summarized the crosstalk associated with Kla, providing a foundation for understanding its potential biological significance.

### Lysine lactylation and lysine acetylation

With the advancement of high-throughput technologies, Kac has emerged as a common PTM that crosstalk with other PTMs. For example, Li et al. found that 75% of Ksucc sites were also acetylated sites in their sequencing analysis of Ksucc and Kac [83]. Moreover, physiological ovarian aging in female mice has also been found to be associated with crosstalk between Kac and other modifications such as Kcr and Kbu [84]. Upon the application of acylation inhibitors, Sir2La was shown to exhibit a slightly higher preference for lysine propionylation (Kpro) over Kac [85]. These findings highlighted the multi-interactive nature of Kac. However, current evidence mainly supported their shared features, while the specific mechanisms underlying the crosstalk between Kla and Kac are unclear. For instance, the Kla sites H3K27, H4K8, H4K12, H2AK11, H2BK15, and H2BK20 are also known acetylation sites [86–88]. Kla and Kac have been shown to influence each other at specific histone sites. For instance, at the H4K12 site, studies demonstrated that this dynamic crosstalk is critical for mouse oocyte meiosis. Specifically, Lin et al. discovered that Tfap2a regulates meiosis in mouse oocytes. Overexpression of Tfap2a upregulated p300, which elevated the levels of H4K12ac and H4K12la, while also increasing H3 K18 la and H4 K16ac, thereby impeding meiosis in oocytes [7]. Moreover, PTM-omics studies have shown that non-histone proteins enriched in Kac and Kla share many common features, with substantial overlap in modification sites. Lin et al. cataloged 2,198 Kac in 925 acetylated proteins and 289 Kla in 181 lactylated proteins in KC. Notably, Kac and Kla showed differential enrichment in distinct subcellular compartments. Furthermore, they identified a Kla site at K82 in the Box-1 protein [89].

Additionally, Kla and Kac utilize shared enzymatic machinery. These enzymes (SIRT3, p300, and CBP) can simultaneously regulate both Kac and Kla, but the precise mechanisms remain unclear [90]. Fan et al. demonstrated that SIRT3 is a key deacetylase in the H4K16 site [80]. Studies demonstrated that SIRT3 shows strong binding affinity and significant deKla efficiency on H4K16la, surpassing other Sirtuins like SIRT1 and SIRT2. Isothermal titration calorimetry (ITC) and HPLC–MS analyses confirmed SIRT3’s superior activity, with its kinetic parameters (KM, Vmax, and Kcat) highlighting its effectiveness. Although HDAC3 was shown to have an even higher deKla activity in vitro, SIRT3 remains particularly potent at the H4K16 site, comparable to its activity in removing β-hydroxybutyrylation at this residue [46].

Moreover, Yang et al. revealed that non-histone Kla can affect Kac at the same site. HMGB1-Kla were mediated by p300/CBP. Lactate promoted HMGB1-Kla, inducing its nuclear-to-cytoplasmic translocation and HMGB1 acetylation, facilitating its cytoplasmic distribution in macrophages, regulated by SIRT1 deacetylase inhibition [80].

However, Kla and Kac are closely related but exhibit notable differences [91]. Although some studies suggested that acetyl-CoA is present in much higher concentrations than L-lactyl-CoA in vivo, it remains unclear under what specific conditions cells activate Kla instead of Kac. Therefore, the correlation of Kla and Kac may be focused in further studies. Therefore, continued exploration of their relationship will be essential for advancing our understanding of Kla-related regulatory mechanisms.

### Lysine lactylation and Ubiquitination (Ub)

Ub, linking its C-terminal glycine (G76) to the ε-amino group of lysine residues on target proteins, typically involved in regulating protein degradation [92–94]. Zhang et al. elucidated that Ub operates downstream of Kla to fine-tune cellular functions. Chen et al. demonstrated that in the context of systemic lupus erythematosus (SLE), lactate facilitates the Kla of cGAS, thereby hindering its interaction with the E3 ubiquitin ligase MARCHF5. This inhibited the degradation of cGAS and elicited a potent IFN-1 response, further underscoring the complexity of Kla in immune regulation [95]. In addition, in esophageal cancer, Li et al. discovered that hypoxia-induced Axin1-K147la promotes its Ub, thereby enhancing the glycolysis and increasing the stemness characteristics of TE1 and EC109 cells. Conversely, overexpression of Axin exhibited a significant tumor-suppressive effect [96].

### Lysine lactylation and phosphorylation

Protein phosphorylation, which typically occurs on serine, threonine, or tyrosine residues, can cooperate with Kla to jointly regulate downstream target genes [97, 98]. Xu et al. discovered that mutations at the S24 site of Sox10 induce alterations in downstream Kla. *Sox*10, a key transcription factor in the SOX family, plays a vital role in neural crest cell and peripheral nervous system development [98–101]. Xu et al. demonstrated that *Sox*10-induced p-Ser and Kla interact, showing that cytoplasmic phosphorylation triggers Kla [81]. They indicated that *Sox*10 is activated by Kla and phosphorylation during vascular smooth muscle cell transformation. The mutation at the S24 site reduced *Sox*10 Kla and phosphorylation, indicating that *Sox*10 Kla relies on S24 phosphorylation. This modification is crucial for *Sox*10 activation and VSMC-to-macrophage-like transformation, providing new insights into cellular changes in vascular lesions.

### Lysine lactylation and Methylation (Me)

m^6^A, an important RNA modification, regulates RNA metabolism and stability [102–104]. In m^6^A-related genes, METTL3, a primary methyltransferase, regulates cell death in various diseases *via* m^6^A modification [82, 105]. Zhang et al. found that METTL3 knockdown alleviates ICH-induced brain injury by regulating Pan Kla. Hemin treatment increased METTL3-Kla, enhancing METTL3 stability which was intensified by lactate [106]. Besides, Gu et al. revealed that histone Kla mediated-ALKBH3 drives tumorigenesis in ocular melanoma by m^1^A demethylation. The upregulation of ALKBH3 is also associated with increased histone Kla, particularly at the H3K18la site. Elevated levels of ALKBH3 in ocular melanoma correlated with reduced m^1^A levels and are linked to poor clinical outcomes. Meanwhile, silencing ALKBH3 resulted in the upregulation of SP100 by removing m^1^A modifications. As can be seen, ALKBH3 is critically involved in the pathogenesis of ocular melanoma, primarily through its regulation of m^1^A methylation [65].

m^5^C has recently been identified to form a regulatory loop with H3K18la. NSUN2, a methyltransferase responsible for m^5^C methylation, has also been established as a “writer” for ENO1. In CRC cells, the accumulation of lactate promoted NSUN2-K356la through H3K18la, a process that is critical for the binding of NSUN2 to its target RNA. This interaction highlights the potential of NSUN2 inhibitors in immune therapy for CRC [107].

### Lysine lactylation and lysine Crotonylation (Kcr)

Kcr and Kla, both metabolite-driven modifications, utilize crotonyl-CoA as their acyl donor [5, 108]. Dai et al. discovered that these two modifications interact with each other, with notable differences observed between late and early neurogenesis. In late neurogenesis compared to early neurogenesis, H3K9cr and H3K18cr levels were elevated. Application of pan-HDAC inhibitors (SAHA and VPA) increased H3K18la levels in P19EC cells, while VPA stimulated H3K14la and H3K18la. These inhibitors also elevated H3K9cr and H3K18cr levels [109]. These results provide new perspectives on epigenetic regulation in neural development and disease processes.

To sum up, the crosstalk between lysine Kla and other PTMs is primarily characterized by competitive occupancy at shared lysine residues, yet gaps remain in heterologous PTMs on distinct proteins or sites. It’s unknown whether it exhibits signal amplification cascades analogous to phosphorylation-mediated networks. Nevertheless, emerging research underscored Kla’s considerable clinical potential, suggesting that mechanistic insights will likely emerge with further investigation. Elucidating Kla-PTM crosstalk mechanisms may address unresolved paradoxes, such as the observation that Kla exerts disproportionately strong cellular effects despite L-lactyl-CoA concentrations being significantly lower than those of classical PTM substrates like acetyl-CoA.

## Biological functions of lactylation

### The role of lysine lactylation in cell survival

Cells respond to various environmental stresses by modulating their physiological states through multiple mechanisms to maintain homeostasis and survival. In addition to classic mechanisms such as proliferation and apoptosis, Kla has been found to be involved in autophagy, ferroptosis, and pyroptosis.

Jia et al. demonstrated a direct link between Vps34 and autophagy, which is crucial for maintaining muscle cell homeostasis [21]. They found that activated ULK1 triggers lactate production *via* LDHA, leading to Vps34-K356la and Vps34-K781la. This modification, mediated by the acetyltransferase KAT5/TIP60, enhanced Vps34’s association with Beclin1, Atg14L, and UVRAG, increasing its lipid kinase activity. Furthermore, Huang et al. demonstrated that TFEB, a key regulator of autophagy, exerts its function through Kla. TFEB-K91la interfered with its interaction with the E3 ubiquitin ligase WWP2, thereby inhibiting TFEB’s Ub and proteasomal degradation. This mechanism further amplified TFEB activity and promoted autophagic flux. Additionally, by using a specific antibody targeting K91-Kla, the authors observed a marked increase in TFEB-Kla levels in clinical PC samples [110]. However, the study did not address whether TFEB-K91la serves as a marker in cancer cells. In cervical cancer, Meng et al. identified that the oncogene DCBLD1 undergoes Kla at the K172 site, which subsequently activates the PPP pathway to drive the proliferation and metastasis of cervical cancer cells.

Ferroptosis is a form of iron-dependent and lipid peroxidation-dependent cell death that typically occurs under conditions of oxidative stress [111–113]. Recent studies by An et al. revealed that tau, a hallmark protein of Alzheimer's disease (AD), is involved in ferroptosis via TAU-K677la. Their findings indicated that mutation at the K677 site inhibited tau Kla, impairing memory and reducing neuronal damage. This effect was mediated by the regulation of iron metabolism factors, such as NCOA4 and FTH1, which attenuate ferroptosis [114].

Non-canonical pyroptosis is a form of programmed cell death mediated by Caspase-11 and GSDMD, typically triggered by cellular infection or injury and accompanied by a robust immune response [115–117]. Li et al. found that lactate regulates Caspase-11-mediated non-canonical pyroptosis during acetaminophen (APAP)-induced acute liver injury by NEDD4-K33la, thereby exacerbating liver damage [118, 119]. Moreover, in a myocardial ischemia–reperfusion (I/R) injury model, Fang et al. discovered that LDHA knockout improved recovery from I/R-induced injury in *vivo*, a process linked to NLRP3 [74]. Further investigation revealed that LDHA promotes NLRP3-K245la, increasing its protein stability, thus unveiling the potential therapeutic role of LDHA in the I/R injury model [120].

### The role of lysine lactylation in cellular development

Multiple studies revealed that histone Kla is associated with early developmental stages and stem cell differentiation, which is crucial for optimizing fertility and stem cell therapies. Decidualization is a crucial event in pregnancy [121, 122]. Zhao et al. identified the H4K12la-HIF1-α-glycolysis loop as a key driver of this process, with significant implications for clinical fertility strategies [53]. Yang et al. highlighted the critical roles of H3K23la and H3K18la in early mouse embryo development [8]. Unlike the stable expression of H3K18ac and H3K23ac, H3K23la and H3K18la levels varied across developmental stages, peaking in blastocysts. Recent studies also revealed a strong connection between H3K18la and osteoblast proliferation. Wu et al. identified that H3K18la plays a significant role in gene regulation in bone marrow stromal cells (BMSCs), especially in osteogenesis and osteoporosis models [14]. In Pkm2^ΔEC^ mouse BMSCs, H3K18la reduction led to the downregulation of 308 genes and upregulation of 35 genes, and *Col1a2*, *Comp*, *Enpp1*, *Tcf7l2* were H3K18la targets involved in bone morphogenesis and mineralization [123]. In parallel, Chen et al. showed that H3K18la regulated *Bmp5*, *Kit*, and *Trpc5* under hypoxia, promoting pulmonary artery smooth muscle cell proliferation [124]. Lin et al. discovered that H4K12la also drove oocyte development. Specifically, Tfap2a overexpression significantly increased H4K12la levels and regulated oocyte maturation [7].

### The role of lysine lactylation in metabolic regulation

The onset of Kla is driven by the upregulation of glycolysis. Numerous rate-limiting enzymes in glycolysis have been implicated in Kla, and the elevation of glycolysis, particularly lactate production, is a defining characteristic of cancer cells. Investigating the Kla-related mechanisms of metabolism-associated enzymes may help elucidate the crosstalk among metabolism, signaling pathways, and epigenetic regulation, and potentially contribute to the development of novel therapeutic strategies for metabolic diseases and cancer.

HK is a key enzyme in glucose metabolism, catalyzing the phosphorylation of glucose to glucose-6-phosphate and ADP, the first and irreversible step in the glycolytic pathway [125–128]. Multiple isoforms of HK exist within cells, including HK1, HK2, HK3, and HK4 (also known as glucokinase) [129]. Research indicated that HK1 and HK2 are influenced by histone Kla, particularly in cancer [130].

The research of HK1 is less extensive compared to HK2, focusing primarily on its role in normal cells [131]. Jiang et al. found that lactate affects glucose uptake, glycolysis, and TCA cycle in NSCLC cell lines BEAS-2B, A549, and H1299, altering metabolic enzyme expression levels. Lactate influenced gene expression by modulating histone Kla at gene promoters, such as downregulating HK-1, G6PD, and PKM transcription while upregulating SDH, IDH, and HIF1-α. ChIP experiments showed that lactate treatment increased histone Kla levels at the promoters of HK1 and IDH3G, suggesting that lactate-induced H4K8la may regulate HK1 transcription [130].Meanwhile, HK2 was recently found to exert its effects *via* Kla rather than acetylation in liver fibrosis and *a-SMA*, *Col1a1*, and *Timp1* were specific targets of Kla [22]. Regrettably, the relationship between HK3, HK4, and Kla has not yet been reported.

Phosphofructokinase 1 (PFK-1), which catalyzes the conversion of fructose-6-phosphate and ATP to fructose-1,6-bisphosphate, is a key rate-limiting enzyme in glycolysis [132, 133]. Wang et al. demonstrated that the role of PFK-1 in bladder cancer is mediated through the regulation of ZEB1-Kla, inhibiting the malignant phenotype of bladder cancer cells. Their study found that downregulation of PFK-1 expression suppresses the proliferation, migration, and invasion of bladder cancer cells. Additionally, PFK-1 was shown to inhibit histone Kla in bladder cancer cells, thereby reducing the transcriptional activity of ZEB1 [134]. These results suggest that PFK-1 could be a novel potential therapeutic target for bladder cancer.

PKM2 is a rate-limiting enzyme in glycolysis, playing a crucial role in converting phosphoenolpyruvate to pyruvate, thereby generating ATP [135, 136]. Wang et al. indicated that PKM2-K62la played a regulatory role in the phenotypic transformation of macrophages. They found that PKM2 expression is significantly elevated in LPS-induced macrophage inflammation models, with lactate playing a critical role. Lactate absence led to PKM2 functional defects. IP-mass spectrometry identified potential Kla sites on PKM2, notably K62, which was confirmed as the primary Kla site through site-specific mutagenesis [137]. Meanwhile, Pan et al. elucidated the glycolysis/H4K12la/PKM2 feedback loop is fundamental to the metabolic reprogramming observed in AD microglia [138].

Specifically, DCBLD1 upregulated the expression and enzymatic activity of glucose-6-phosphate dehydrogenase (G6PD), significantly boosting PPP activity. This regulatory mechanism relied on HIF-1α enrichment at the DCBLD1 promoter region, which enhanced DCBLD1 mRNA expression. Furthermore, lactate-induced DCBLD1-Kla stabilized its expression [73]. Similarly, enzymes of the TCA cycle, like citrate synthase, and those involved in OXPHOS, such as PDHA1 and CPT2, are also regulated by Kla [139]. Hypoxia induced the accumulation of AARS2, leading to PDHA1-K336la and CPT2-K457la, which subsequently resulted in alterations of OXPHOS.

### The role of lysine lactylation in immune response

In both cancer immunity and microbiome immunity, studies have shown that Kla is a key regulatory mechanism, whether in histones or non-histones.

H3K18la plays a crucial role in the recruitment of inflammatory cells, maintenance of immune responses, and drug resistance. CXCL1 and CXCL5 are neutrophil chemokines that regulate neutrophil migration and activation through interactions with the CXCR2 receptor [140–142]. Zhou et al. found that GPR37 regulates the expression of CXCL1 and CXCL5 by enhancing H3K18la, thus promoting neutrophil recruitment [143]. Rela and NF-κB1, key components of the NF-κB signaling pathway, encode the p65 and p50 proteins, respectively, and are crucial for maintaining immune responses [144–146]. Wei et al. discovered that H3K18la enhances Rela and NFκB1 promoter binding, stimulating NF-κB signaling in an AD model [147]. In addition, H3K18la is correlated with drug resistance, a critical challenge in treating inflammatory responses. Ma et al. demonstrated that H3K18la is involved in the treatment of methylsulfonylmethane (MSM) in methicillin-resistant Staphylococcus aureus (MRSA) by increasing H3K18la in macrophages and promoting macrophage polarization to the M2 phenotype, thus enhancing Arg1 expression and offering protective effects in MRSA-infected animal models [148]. In 2019, Zhang et al. identified an increase in Kla during M1 macrophage polarization, heralding the advent of immunometabolism research [149]. As this burgeoning field continues to evolve, the pivotal role of H3K18la within it warrants deeper investigation. Wang et al. found that mastitis inflammation is also associated with H3K18la, which activated the TLR4/NF-kB signaling pathway [150].

Wang et al. first demonstrated that Mecp2-K271la in endothelial cells slows the progression of atherosclerotic cardiovascular disease (ASCVD) [77]. Mecp2-K271la inhibited the transcription of epiregulin (*Ereg*) in endothelial cells, reducing phosphorylation of epidermal growth factor receptor (*Egfr*) and mitogen-activated protein kinase (MAPK) activity. This modulation decreased the expressions of *Vcam*−1, *Icam-1*, *Mcp-1*, *Il-1β*, *Il-6*, and *Enos*, thereby slowing the development of ASCVD. Besides, BCAP is a signaling adaptor protein initially identified for its role in PI3K signaling [151]. Irizarry-Caro discovered that BCAP links Toll-like receptor (TLR) activation to glycolytic metabolism, regulating intestinal inflammation and tissue repair processes. In BCAP-deficient macrophages, there is a significant reduction in glycolysis and lactate production, accompanied by decreased Pan Kla levels. Exogenous lactate supplementation restored Pan Kla levels in BCAP-deficient macrophages, highlighting the crucial role of glycolysis and lactate production in cellular metabolic regulation and subsequent immune functions [152].

## Diseases associated with lactylation

### The role of lysine lactylation in cancer

**Fig. 5:**
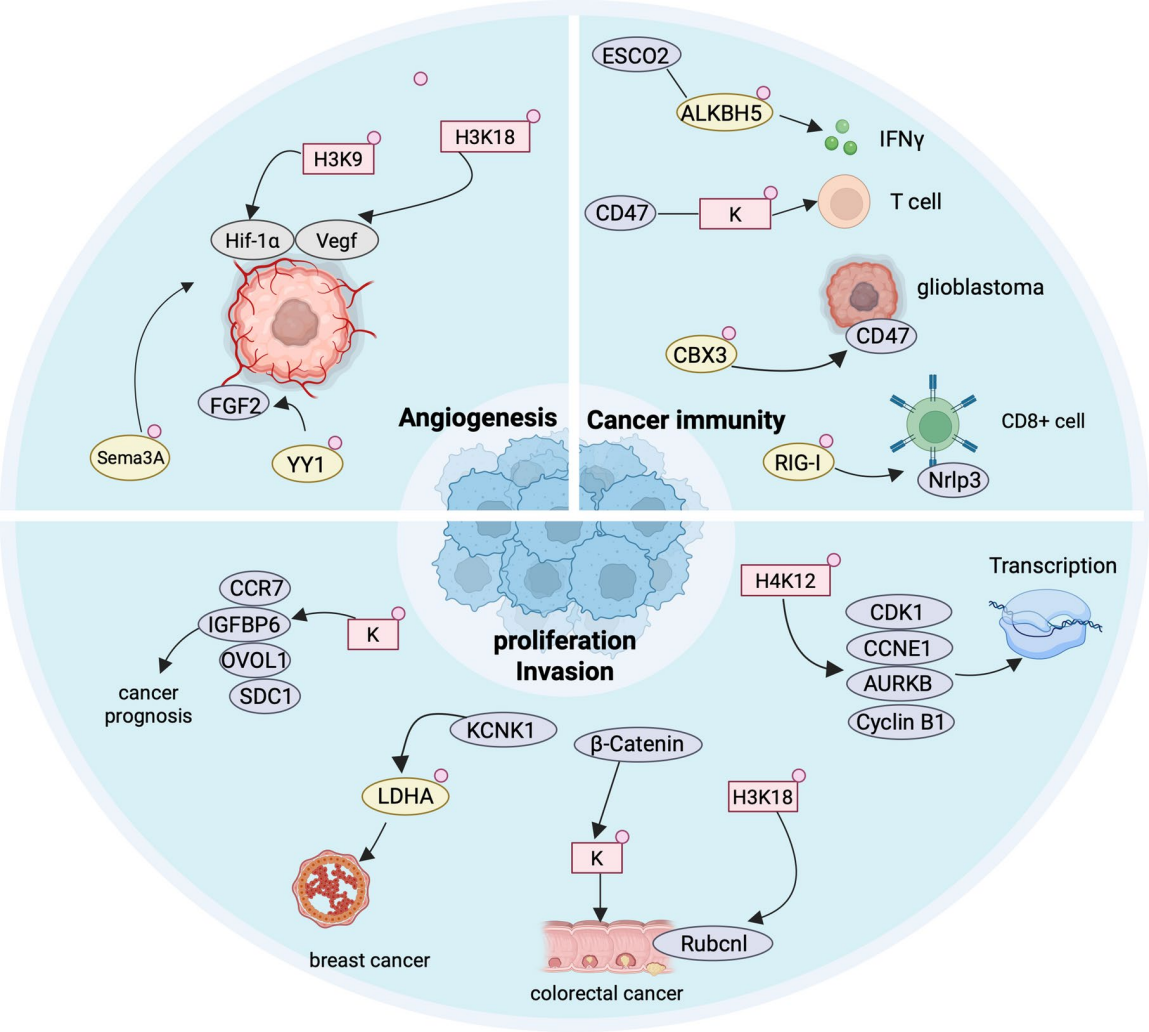
The roles of histone lactylation and non-histone lactylation in cancer. Histone Kla regulates tumor angiogenesis by affecting the levels of downstream HIF1-α and Vegf. In non-histone proteins, YY1-Kla influences FGF2, and Sema3A-Kla affects HIF1-α, both contributing to the regulation of angiogenesis in cancer cells. In tumor immunity, histone Kla can impact T cell immune responses, while ALKBH5-Kla, CBX3-Kla, and RIG-I-Kla participate in tumor immunity by modulating the levels of IFN-γ, CD47, and NLRP3. In the regulation of cancer cell proliferation, histone Kla affects tumor growth through CDK1, CCNE1, AURK, Cyclin B1, CCR7, IGFBP6, DVOL1, and SDC1, whereas LDHA-Kla influences breast cancer proliferation

In a comprehensive lactylome analysis of HCC, Zhang et al. mapped 9,275 Kla sites in tumors and adjacent liver tissues, with 9,256 of these sites localized on non-histone proteins [18]. They underscored the central role of Kla as a critical modification in cancer. In 2011, Hanahan and Weinberg proposed the ten hallmarks of cancer [153]. Current research showed that Kla is closely associated with several other hallmarks, including the tumor microenvironment (TME) remodeling, angiogenesis, tumor immunity, tumor growth and invasion (Fig. 5). In this section, we provide a comprehensive summary of the features of Kla in the cancer.

#### Angiogenesis

Angiogenesis is one of the hallmarks of TME, with VEGF being the most prominent known angiogenic factor [154–156]. Fan et al. discovered that the angiogenic effect of VEGF in endothelial cells is linked to the interaction between H3K9 la and HDAC2. They suggested that disrupting the H3K9la-HDAC2 feedback loop could be a novel approach for anti-angiogenesis therapy [54]. Additionally, YY1 has also been shown to regulate angiogenesis through Kla. YY1 is a multifunctional transcription factor that can repress, activate, or initiate transcription depending on promoter structure and cellular context [157, 158]. Wang et al. demonstrated that YY1 alters chromatin structure and DNA conformation, thereby affecting gene expression [24]. Under hypoxic conditions, increased lactate production in microglia upregulated the expression of acetyltransferase p300, leading to the YY1-K183la. This modification accelerated FGF2 mRNA transcription and expression, thereby enhancing angiogenesis in endothelial cells and potentially contributing to pathological changes. Anti-angiogenic therapies face challenges such as drug resistance [159]. Luo used the anti-angiogenic drug KIAA1199 for prostate cancer and found it to be positively correlated with HIF-1α overexpression and angiogenesis markers. Further experiments confirmed that HIF-1α is a transcriptional activator of KIAA1199, regulating lactate transport in PC cells through Kla [160]. This also suggests that HIF-1α may be a potential target for anti-angiogenic therapy. Yu et al. found that H3K18la regulates Sema3 A (a tumor suppressor and angiogenesis inhibitor) transcription in PC cells, where evodiamine impaired HIF-1α-mediated H3K18la, reducing Sema3A-mediated angiogenesis and PD-L1 expression, and inducing ferroptosis [161]. These studies provided a theoretical basis for the development of novel anti-angiogenic therapeutic strategies.

#### Tumor immunity

Tumor immunity is a hallmark of cancer, and its underlying mechanisms are linked to Kla [162–164]. CD8^+^ T cells are critical effector cells in tumor immunity [162, 165–167]. Raychaudhuri et al. showed that the activation and function of CD8^+^ T cells are regulated by H3K18la and H3K9la. In their preclinical model, they found that targeting H3K18la and H3K9la alters the antitumor immune response mediated by CD8^+^ T cells [168]. Additionally, Kla has been shown to contribute to immune evasion in glioblastoma (GBM). Wang et al. found that the “writer” p300, in collaboration with the heterochromatin component chromobox protein homolog 3 (CBX3), promoted the production of L-lactyl-CoA, which in turn enhanced the release of CD47, a “don’t eat me” signal, from GBM cells [169]. Additionally, Xiong et al. discovered that Kla-driven METTL3 enhances TME’s immunosuppressive capacity through RNA m^6^A modification. Specifically, exogenous lactate elevated Pan Kla and H3K18la levels in alveolar epithelial cells (AECs) while inhibiting lactate transport reduced these levels [63]. Then, elevated H3K18la upregulated METTL3 in TME, which are crucial for binding target RNA [170]. Meanwhile, Regulatory T (Treg) cells and CD8^+^ T cells are key effector cells in the TME. Gu et al. found that *E. coli* enhances the lactate-RIG-I-Kla-Nlrp3 interaction, and the deletion of Nlrp3 affects the immunosuppressive activity of Tregs and the antitumor activity of CD8^+^ T cells. Surprisingly, the authors screened 220,000 compounds and identified a drug targeting the RIG-I Kla site, which enhances the sensitivity of colorectal liver metastasis (CRLM) to 5-fluorouracil (5-FU), further supporting Kla as a potential therapeutic target [171]. Additionally, Tong et al. discovered that Kla plays a crucial role in the innate immune response triggered by HSV-1, KSHV, and MPXV. They identified an innate immune-regulating protein, AlkB homolog 5 (ALKBH5). Viral infections increased the binding of ALKBH5 to the acetyltransferase ESCO2. Overexpression of ESCO2 enhanced the Kla of ALKBH5, thereby promoting the biogenesis of IFN-β mRNA [172]. This discovery highlights the regulatory role of Kla in innate immunity. Leo et al. found that the loss of antitumor immunity in GBM is caused by an increase in monocyte-derived macrophages (MDMs). In MDMs, the deletion of PERK led to the elimination of histone Kla, which promoted the accumulation of intratumoral T cells and delayed tumor growth. Excitingly, PERK deletion, when combined with immunotherapy, effectively inhibited the GBM progression [173]. This provides further support for Kla as a therapeutic target. In addition to T cells, tumor immunity related to CD71^+^ neutrophils is also associated with changes in their Kla levels [174].

#### Tumor growth and invasion

Uncontrolled proliferation and invasive capability are also classic hallmarks of tumors [175–177]. Miao et al. found that Kla regulates β-catenin-induced malignant proliferation in CRC cells [178]. In addition, Cheng et al. found that H3K18 la promotes high expression of *Rubcnl* in CRC by positively regulating its transcription, which supported cancer cell proliferation and survival, resulting in poor CRC prognosis and reduced bevacizumab efficacy [179]. In breast cancer, Hou et al. found that the potassium two-pore domain channel subfamily K member 1 (KCNK1) is not only linked to poor prognosis in patients but also drives malignant proliferation of cancer cells in vitro by regulating LDHA-Kla. The authors also proposed that KCNK1 could be a potential biomarker for breast cancer [180]. Additionally, after constructing a model linking GC to Kla, Yang et al. found a strong association between Kla and the progression and survival rates of GC, indirectly highlighting the role of Kla [181]. In HCC, Wu et al. investigated the relationship between Kla-specific genes and cancer prognosis, identifying core genes NR6A1, OSBP2, and UNC119B as potential new therapeutic targets for HCC [182]. Lately, Wang et al. primarily found that the oncogene BRAF^V600E^ promoted anaplastic thyroid carcinoma proliferation by increasing lactate and altering H4K12la expression. Specifically, H4K12la activated proliferation-related genes (CTGF, CCNE1, CDK1, KLF2, IL1B, and AURKB). In the 8503c cell line, ChIP-seq showed H4K12la enrichment at these gene promoters. BRAF^V600E^ inhibitor PLX4032 reduced H4K12la at CDK1, CCNE1, and AURKB promoters, consistent with mRNA expression changes [183]. Cell cycle dysregulation is also linked to H4K12la, which contributes to the progression of non-small cell lung cancer (NSCLC). They indicated that H4K12la promotes chemotherapeutic drug resistance by promoting transcriptional activation of the Cyclin B1 (CCNB1) gene [69]. Beyond cell cycle, H4K12la is also closely linked to chronic diseases such as atherosclerosis and renal fibrosis, underscoring the necessity of exploring its underlying mechanisms [184, 185].

### The role of lysine lactylation in cardiovascular pathobiology

In the cardiovascular system, Kla orchestrates pathophysiological processes through multi-dimensional molecular mechanisms, with its pivotal role manifested in maintaining myocardial structural and functional homeostasis [186]. Studies demonstrated that Kla stabilizes interactions between α-myosin heavy chain (α-MHC) and titin, essential for sarcomere integrity [187]. For instance, loss of α-MHC- K1897la directly disrupted sarcomere stability, accelerating heart failure progression. Notably, Kla exhibits dual regulatory capacity in inflammation and fibrosis. During early myocardial infarction, H3K18la activated monocyte repair genes (VEGFA and IL-10) to attenuate inflammation and promote angiogenesis [188], whereas excessive lactate accumulation may drive pathological cardiac fibrosis via Snail1-Kla mediated TGF-β pathway activation [187].

During infarct repair, histone H3K18la enhanced vascular regeneration and anti-inflammatory responses through repair gene activation (LRG1 and VEGFA), with therapeutic modulation achievable *via* targeting GCN5 or LDH inhibitors (FX-11) [188]. Intriguingly, Kla’s effects in atherosclerosis displayed cell type specificity. MECP2-K271la suppressed inflammatory cytokine secretion (IL-6 and MCP-1) to stabilize plaques [19], while vascular smooth muscle cell H4K12la potentially accelerated the plaque progression by inducing senescence-associated secretory phenotype (SASP) [184]. These findings underscore Kla’s dualistic “repairer-destroyer” roles in cardiovascular diseases, with spatiotemporal modification patterns offering critical insights for targeted therapeutic development.

### The role of lysine lactylation in neurological disorders

Emerging evidence has established that Kla plays complex yet pivotal roles in the pathogenesis of neurological disorders. For example, Alzheimer’s disease (AD) progression is closely linked to metabolic dysregulation in microglial cells. Elevated H4 K12 la levels are observed in AD brains, particularly in microglia surrounding β-amyloid (Aβ) plaques, where this modification activates glycolysis-related genes (PKM and LDHA), forming a “glycolysis-histone lactylation-PKM2” positive feedback loop that exacerbates lactate accumulation, neuroinflammation, Aβ deposition, and cognitive decline [11]. Similarly, traumatic brain injury (TBI) exhibited lactate induced TUFM-Kla, which disrupted TOMM40 interaction, impaired mitophagy, and promoted neuronal apoptosis [189]. Parkinson’s disease involved Kla-mediated modulation of mitochondrial protein SLC25A4, SLC25A5 and SlC7A11 stability, while cerebral infarction showed LCP1-Kla aggravating ischemic damage *via* neuronal apoptosis [190–192].

Neuroinflammatory mechanisms and peripheral nerve injury are also dependent on Kla. Peripheral nerve damage induced Schwann cell lactate dysmetabolism, driving neuronal lactate overload, oxidative stress, and axonal degeneration [193]. Glioblastoma microenvironmental lactate promoted IL-10-Kla and LCP1-Kla to suppress T-cell activity, facilitating immune evasion and enhancing tumor invasiveness [194]. Moreover, Hu et al. demonstrated that H4K12la plays an important role in microglia-mediated tissue repair [195]. Metabolic crosstalk with Kla offered mechanistic insights. They found that lactate shuttling fueled neurons, while Kla regulated energy dynamics *via* LDHA and PDK1. Hypothalamic POMC neuron FAM172 A deficiency enhanced H4K12la to stimulate α-MSH synthesis and ameliorate obesity-related metabolic disorders [196]. Schizophrenia models revealed aberrant H3K9la and H3K18la elevation upregulating HMGB1 to induce hippocampal neuron apoptosis, suggesting Kla mediated synaptic plasticity impairment [197]. Though its shuttle mechanism had not been conclusively demonstrated prior to the discovery, Kla is serving as the crucial metabolic fuel and signaling molecule in the nervous system [198].

### The role of lysine lactylation in metabolic disorders

Diabetes is one of the most common metabolic disorders, and Kla plays a crucial regulatory role in it [199–201]. Chen et al. found that 356 lysine Kla sites on 165 proteins were increased in kidney samples from patients with diabetic kidney disease (DKD). Subcellular analysis revealed that 269 of these sites were in mitochondria. They further discovered that ACSF2-K182la causes mitochondrial dysfunction, which contributed to the development of diabetes. Elevated lactate levels in urine are associated with the progression of DKD [202]. Zhang et al. found that histone Kla promotes KLF5 expression, thereby accelerating the epithelial-mesenchymal transition, which worsens DKD [203]. Obesity and insulin resistance are common manifestations of metabolic disorders [204]. Maschari found that Kla is linked to insulin resistance and obesity. Sodium lactate can activate Kla and IRS-1 serine 636 phosphorylation in human skeletal muscle cells. In contrast, inhibiting lactate levels showed the opposite effect. Diabetic retinopathy (DR) is another common complication of diabetes caused by metabolic imbalance [205–207]. Xue et al. found that Kla drives changes in Fat Mass and Obesity-Associated Protein (FTO), and inhibition of FTO with FB23-2 suppressed angiogenesis associated with retinopathy [208]. Gestational diabetes mellitus (GDM) is also a common metabolic disorder [72, 209]. Huang et al. discovered that Kla levels were significantly elevated in GDM patients, and combined RNA-seq and ChIP-seq analyses identified CACNA2D1 as a key regulatory protein for histone Kla in GDM [71]. Furthermore, diabetic cardiomyopathy (DCM) is a major cause of death among diabetic patients. Ma et al. found that lactate is significantly elevated in type 2 diabetes patients and MCT4 was highly expressed in DCM, then enhancing H4 K12 la-induced inflammatory infiltration [210]. Alternatively, mitochondrial dysfunction is also associated with metabolic disorders [211]. Yu et al. demonstrated that H3 K18 la regulates the inner mitochondrial membrane transporter SLC25A29 [212]. Unfortunately, the function of Kla in key metabolic tissues such as adipose tissue, liver, and pancreatic beta cells remains poorly understood.

### The role of lysine lactylation in other diseases

Kla is involved in a wide range of diseases. In addition to cardiovascular, neurological, cancer, and inflammation-related conditions, it has also been shown to be associated with various congenital developmental disorders. Du et.al found that *P. gingivalis* promotes the transcription of ADAM17 by activating glycolysis and H4K12la, thereby inducing macrophage efferocytosis and impairing osteogenesis of palatal mesenchymal stem cells, ultimately affecting palatal shelf fusion and bone formation [213]. This is the first evidence linking Kla to cleft palate, highlighting the necessity of further Kla research.

Additionally, Xu et al. identified H3K23la as a key player in the pharmacological action of Gegen Qinlian decoction (GQD) in UC. GQD reduced lactate production and downregulated Pan Kla and specific sites like H3K18la, H3K23la, H4 K8 la, and H4 K12 la in a UC model, an effect reversible by exogenous lactate [214]. Kla may also be a feature of critical illness. Chu et al. found that the level of H3 K18 la was higher in critically ill patients compared to healthy controls, and it was associated with the elevation of several critical illness markers [68]. Beyond above diseases, recent studies indicated that the pathological spectrum associated with Kla is continuously expanding. For instance, Ju et al. demonstrated that H4 K12la is involved in collagen synthesis. Their findings suggested that the lactate-H4K12la-HDAC3-TGF-β axis may serve as a novel strategy to combat cellular senescence [215]. In addition, Kla played a significant role in processes such as cartilage development, intervertebral disc degeneration, and cochlear development [216–218]. With the ongoing advancement of research in this field, the underlying mechanisms of these related diseases are expected to be further elucidated.

## Therapeutic implications of lysine lactylation

Kla has gradually emerged as a therapeutic target in the context of clinical disease research. Among current strategies, modulation of Kla is commonly achieved by targeting lactate metabolism. As key regulators of lactate shuttling, MCT1 and MCT4 have attracted particular attention. Targeting Kla has been shown to improve outcomes in central nervous system diseases, ocular diseases, and immune dysregulation in psoriasis [219–222]. Targeting LDH is also a major approach to inhibit lactate production. In the PM2.5-induced lung inflammation and fibrosis model, Li et al. found that Kla is involved in the pathogenesis. The LDH inhibitor GNE-140 alleviated PM2.5-induced lung inflammation and fibrosis in mice by inhibiting glycolysis and subsequent histone Kla [223]. However, targeting lactate transport is an indirect way to regulate Kla and may trigger many nonspecific effects beyond Kla.

The increasing number of studies have shown that targeting enzymes involved in Kla is a more precise strategy for regulating Kla. For example, targeting p300/CBP has been shown to alter both histone and non-histone Kla [224]. Recently, targeting Sirt3 was also found to modulate the expression level of H3K9la in esophageal cancer [225]. Additionally, KAT8’s role in CRC suppression, the tumor resistance mechanisms associated with NBS1 K388 blockade, and the enhanced tumor-suppressive effects observed with the combination of Sirt2 inhibitors and Elesclomol have also been highlighted. Studies have also shown that combined inhibition of CBP and LDH in Kla pathways may restore chemotherapy sensitivity [77]. However, since these enzymes are shared among various acylation modifications, such interventions may cause many other nonspecific effects. Recent research on Kla-specific enzymes may represent an important breakthrough in addressing this issue. Zhu, Chen, Xie, and Sun et al. demonstrated that the ACSS2-KAT2A interaction blocking peptide, when combined with anti-PD-1 antibodies, exerts superior tumor-suppressive effects. This approach not only enables specific targeting of Kla, but also shows improved therapeutic effects against cancer when combined with immunotherapy.

In addition to being a therapeutic target, Kla has also gradually emerged as a biomarker in various diseases. For example, RCCD1 may play an important role in immune infiltration in lung adenocarcinoma. Besides, research showed that Kla is associated with several oncogenes, including the tumor suppressors CCR7 and IGFBP6, and the oncogenes NDUFAF6, OVOL1, and SDC1. LDH inhibitors effectively reduced Kla levels [226]. However, these studies are still at a preliminary stage, and it is believed that Kla will yield more promising results in precision therapy in the future.

## Discussion and perspectives

Since its discovery in 2019, Kla has played significant roles in key biological processes, such as macrophage polarization, autophagy, stem cell reprogramming, and proptosis. Within just the past year, several key questions surrounding Kla have been addressed. Mechanistically, distinctions among K_L-la_, K_D-la_, and K_ce_ isomers have been clarified, AARS1/2 have been shown to directly utilize lactate as a donor to catalyze Kla, and GTPSCS and ACSS2 have been identified as L-lactyl-CoA synthetases. Functionally, p53-K120la has been implicated in tumor promotion, while MRE11-K673la contributes to chemoresistance. On the therapeutic front, AT-101 derivatives have been found to block lactate production and transport, and targeting AARS1-KAT8 has been shown to reverse drug resistance. Such rapid progress underscored the dynamic nature of Kla research and its relevance across multiple disease contexts.

However, there are some challenges. Although D-lactate has been shown to lead to K_d-la_, its universality remains unclear, and the underlying mechanism from D-lactate to K_d-la_ has yet to be elucidated. In addition, early studies on K_l-la_ predominantly focused on common acyltransferases like p300 and P53. Recent studies have expanded the repertoire to include enzymes such as GNAT13, HBO1, YiaC, TIP60, NBS1, AARS1 and KAT2A. This broadens the scope of research but also introduces complexity and challenges for comprehensive characterization. Many new “writers” have been discovered through the study of non-histone Kla, which could be a promising direction for future researchers.

Furthermore, among the enzymes involved in the Kla process, “writers” have received the most attention, followed by erasers, while readers have been the least studied in current research. However, reader could be a significant breakthrough in identifying specific targets for Kla. Unlike certain “writers” and erasers with broader applicability, the reader exclusively transmits Kla signals to downstream genes. Identifying a Kla-specific reader could also facilitate the clinical application of Kla.

Moreover, lysine residues undergo various PTMs, including acetylation, methylation, and ubiquitination, leading to potential competitive interactions. However, the competitive interaction between different modifications at the same site is still poorly understood. For instance, the Kla sites H3K27, H4K8, H4K12, H2AK11, H2BK15, and H2BK20 are also known acetylation sites. Existing literature primarily focused on the concurrent expression of both modifications at these sites without providing in-depth insights into how these modifications compete or collaborate. Future researchers, when observing multiple modifications at the same site, could attempt to explore which acetylation modification under the same conditions as the primary mechanism affecting the phenotype by adding different substrates. This approach may help to refine the understanding of the mechanisms underlying acetylation modifications. This also helps identify specific targets of Kla, facilitating the development of future clinical applications.

Despite the challenges, Kla research holds the potential to provide significant insights and innovations in translational medicine.

## Data Availability

Not applicable.
